# The smell of death. State-of-the-art and future research directions

**DOI:** 10.3389/fmicb.2023.1260869

**Published:** 2023-09-14

**Authors:** Julia Cieśla, Julia Skrobisz, Bartosz Niciński, Magdalena Kloc, Katarzyna Mazur, Artur Pałasz, Gulnaz T. Javan, Marcin Tomsia

**Affiliations:** ^1^Faculty of Medical Sciences in Katowice, Medical University of Silesia, Katowice, Poland; ^2^Department of Histology, Faculty of Medical Sciences in Katowice, Medical University of Silesia, Katowice, Poland; ^3^Department of Physical and Forensic Science Programs, Alabama State University, Montgomery, AL, United States; ^4^Department of Forensic Medicine and Forensic Toxicology, Faculty of Medical Sciences in Katowice, Medical University of Silesia, Katowice, Poland

**Keywords:** odor mortis, volatiles, organic compounds, forensic analysis, mercaptans

## Abstract

The decomposition of a body is inseparably associated with the release of several types of odors. This phenomenon has been used in the training of sniffer dogs for decades. The odor profile associated with decomposition consists of a range of volatile organic compounds (VOCs), chemical composition of which varies over time, temperature, environmental conditions, and the type of microorganisms, and insects colonizing the carcass. Mercaptans are responsible for the bad smell associated with corpses; however, there are no unified recommendations for conducting forensic analysis based on the detectable odor of revealed corpses and previous research on VOCs shows differing results. The aim of this review is to systematize the current knowledge on the type of volatile organic compounds related to the decomposition process, depending on a few variables. This knowledge will improve the methods of VOCs detection and analysis to be used in modern forensic diagnostics and improve the methods of training dogs for forensic applications.

## 1. Introduction

Death is a multifaceted process, wherein each individual cell and tissue has its own metabolic rate and cessation time. The termination of functions of specific cells is a hallmark of death, defined by the dying process. On the other hand, some cells continue to present vital signs, e.g., movement or the ability to be cultured, for minutes, hours, or even days after somatic death. Consequently, if the demise of cells in a peculiar system does not occur simultaneously, *postmortem* changes and the freeing of volatile organic compounds that contribute to odor mortis will fluctuate in terms of time, intensity, and profile. Scientific evidence verifies undoubtedly that irreversible cardiac arrest and the succeeding ceasing of cellular metabolic functions are the commencement of decomposition’s changes (Payne-James and Jones, 2019). The processes following the cessation of vital functions determine the profile of released volatile compounds that can be analyzed. However, environmental, individual, and many other factors also affect the VOC profile. The combination of each characteristic and the interaction between each factor renders the research of volatile profiles extraordinarily complex and difficult. There are few articles on the profile of VOCs released during the decomposition of the body. Moreover, cadaverine and putrescine are primarily responsible for the distinct smell of death, but they are not volatile organic compounds.

However, the development of forensic medicine has caused the need to search for new methods to determine the postmortem interval, cause of death, and its circumstances as precisely as possible. For this reason, the analysis of volatile organic compounds, which undoubtedly make up the overall characteristics of the body after death, can provide new information to complement the data obtained by standard autopsy.

The aim of the review is a systematic and critical analysis of the current state of knowledge on the profile of VOCs emitted from decomposing material. Due to the heterogeneity of deaths and clear differences in the detected VOCs profiles, the factors influencing the types of detectable volatile compounds have been systematized. Recent research suggests that bacterial colonies and insects inhabiting decomposing corpses can also significantly influence the type of VOCs detected, as summarized in the review. Particular attention was paid to the practical aspects of knowledge about volatile compounds, through an in-depth analysis of the role of dogs and other animals whose outstanding sense of smell and ability to detect VOCs is used in forensics, and the latest research on portable VOCs detectors and analysis methods for research and diagnostic purposes was summarized.

## 2. *Postmortem* biochemical changes

The discussion surrounding *postmortem* processes assumed a more comprehensive approach with the introduction of the term “taphonomy” in the 1940s. Forensic taphonomy encompasses various domains such as forensic medicine, archeology, and paleobiology (Pokines et al., 2021) thus it can be regarded as the interdisciplinary study of the physiological alterations that occur in an organism after death (Schotsmans and Márquez-Grant, 2017). To enhance comprehension of the phenomena associated with human decomposition and *postmortem* body changes, the concept of *postmortem* interval (PMI) is paramount. Throughout history, multiple techniques have been engaged to assess PMI. One of the earliest methods involved determining the time of death by analyzing the rate of morphological changes, dating back to the third and fourth century BC. Subsequently, the examination of DNA degradation and potassium levels in the vitreous humor has been utilized to estimate the PMI, although nowadays their practicality is limited to the early stages of decomposition (Donaldson and Lamont, 2013). The potential relevance of the chronological sequence and progression of insect colonization on decomposing human remains was also investigated. Nevertheless, the colonization pattern of necrophagous insects is subject to weather and environmental variations (Matuszewski, 2021). Nowadays, advanced techniques for calculating PMI are associated with enhanced exactness in determining the time of death. Current evidence indicates that PMI estimation based on soil matrix analysis of the released fluids, molecular biology methods, RNA analysis, tissue nano-mechanics, and value of Accumulated Degree-Days has the potential to provide the most precise reflection of *postmortem* body alterations, encompassing both the early and advanced stages of decomposition (Megyesi et al., 2005; De-Giorgio et al., 2019; Scrivano et al., 2019).

Decomposition is an integral aspect of forensic taphonomy, and it typically commences, on average, 4 minutes after death, persisting until skeletonization occurs (Vass, 2001; Pokines et al., 2021). Throughout this process, the corpse undergoes numerous chemical and physical changes influenced by biotic and abiotic factors, resulting in corporal modifications of appearance and the emission of odor (Goff, 2009; Verheggen et al., 2017). All these transformations reflect the process of reverting “from dust to dust,” wherein macromolecules of the body, including proteins, carbohydrates, sugars, collagen, and lipids, are broken down into simpler molecules and gasses (Vass, 2001; Deo et al., 2020). The percentage distribution of body macromolecules is individual-specific and subordinate to genetic factors, leading to a variation in the cadaveric volatiles released from remains (Verheggen et al., 2017; Deo et al., 2020; Raymer et al., 2020). A simplified categorization of the decomposition process consists of five stages: fresh, bloated, active, advanced, and dry (Carter et al., 2007; Statheropoulos et al., 2011; Raymer et al., 2020). However, there is no standardized classification of the stages of decomposition due to differences in geographic location, weather conditions, scavenger activity, and other factors affecting the rate and characteristics of decomposition (Dent et al., 2004; Goff, 2009).

The first stage of early decomposition is referred to as ‘fresh’. Vivid human cells immediately became poisoned by increasing amounts of carbon dioxide in the blood and accumulated waste products because of the deprivation of oxygen. This stage is characterized by the progressing degradation of soft tissue and enzymatic cell digestion, a process titled autolysis (Vass, 2001; Dent et al., 2004; Raymer et al., 2020). As evidence of this phase can be distinctive chronologically early *postmortem* changes. One such change is the onset of *pallor mortis*. This most rapid effect is caused by the cessation of blood flow through the skin capillaries typically occurs within 20 min *postmortem* (Schäfer, 2000; Onyejike et al., 2022). Subsequently, ‘the cooling of the body to ambient temperature’ defined as *algor mortis* proceeds (Schäfer, 2000; Gonçalves, 2022; Onyejike et al., 2022). Taking into consideration the opacity of the cornea, the absence of ocular changes indicates that death happened within the preceding 2 hours (Wróblewski and Ellis, 1970; De-Giorgio et al., 2021). The settling of immobile blood to the lowest parts of the body due to the gravitational force, resulting in visible skin discoloration, is described as *livor mortis. Rigor mortis*, the last discernable key indicator of death, arise due to the depletion of ATP and accumulation of lactic acid in the muscle cells, leading to a rigid connection between myosin and actin (Kori, 2018; Titus et al., 2022). Beyond that ‘fresh’ stage lasting approximately one to three days, there are no other distinctive features. The body does not exhibit any specific discoloration, the odor is subtly perceptible, and insects do not display activeness, with only blowflies and wasps being detectable within minutes after death (Carter et al., 2007; Moraleda et al., 2022).

Autolysis shall be preceded by putrefaction – the ‘bloat’ stage lasts from three to seven and ten days after death (Statheropoulos et al., 2011). Following cellular acidification, the nutrient-rich components are released by cells, forming the medium for microbial communities. Anaerobic bacterial fermentation is associated with the breakdown of the soft tissues into gasses (hydrogen sulfide, carbon dioxide, methane, ammonia, sulfur dioxide, and hydrogen), liquids (e.g., organic acids), and simple molecules which suggest the initiation of the ‘bloated’ stage. Among noticeable signs of the following stage can be distinguished: bloating, discoloration of sulfhemoglobin (the gaseous H2S combines with iron), marbling, skin slippage, development of fly larvae, and increasing temperature (Vass, 2001). Finally, the strong odor becomes noticeable. The early decay phase stops after body deflation. Successively alkaline liquids made by putrefaction attract multiple organisms closely related to the sophisticated and odorous decay phases (Vass, 2001; Dent et al., 2004; Goff, 2009; Statheropoulos et al., 2011; Kori, 2018; Moraleda et al., 2022).

**Figure 1:**
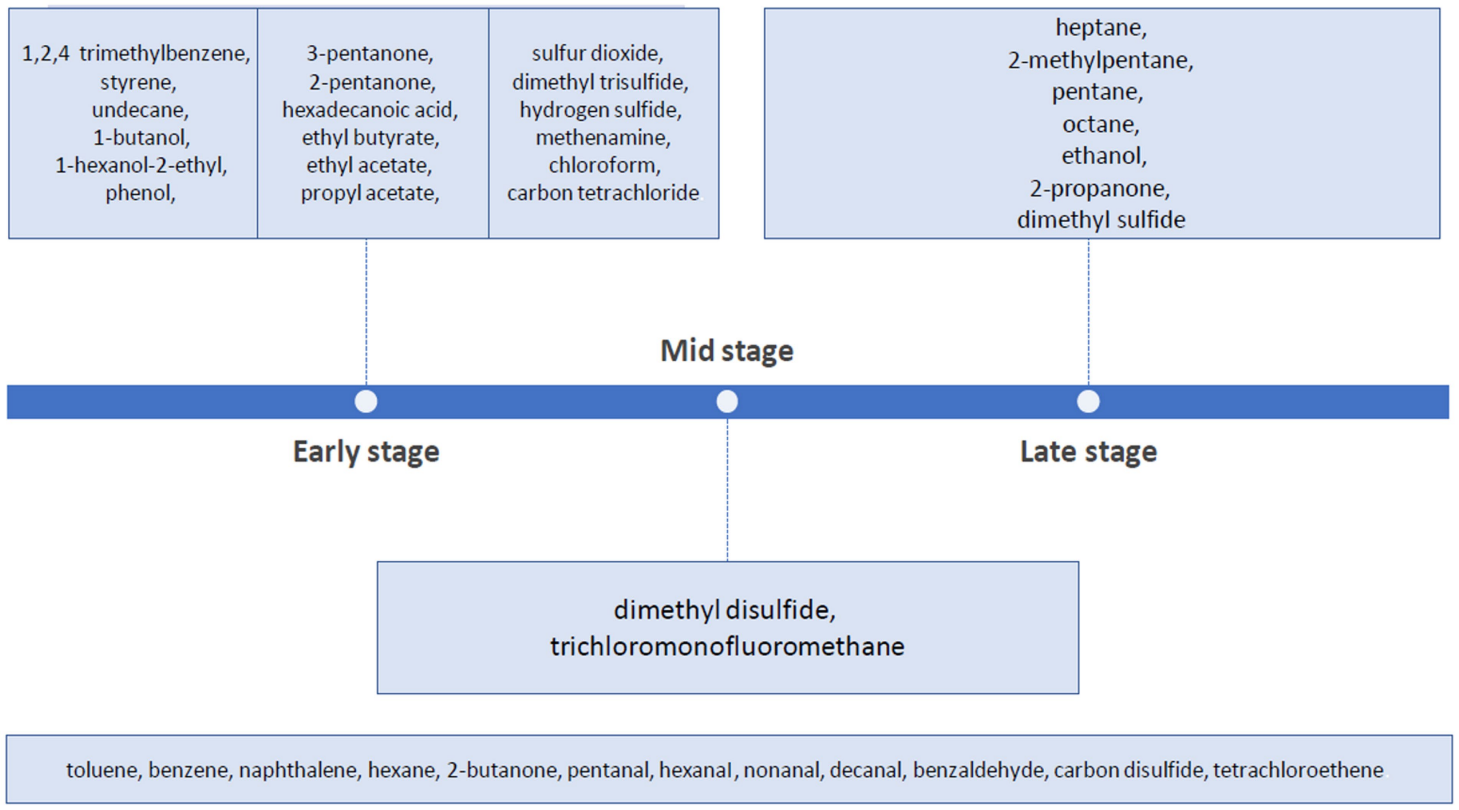
Characteristics of secreted chemical compounds during particular phases of body decomposition.

Active decay begins between seven and ten days and lasts approximately until 20 days. Maggots (*Calliphoridae larvae*), many insect families demonstrate their peaks of activity pursuing a massive breakdown of protein and fat into amino acids, skatole and indole, cadaverine, putrescine, and many other products. As a result, soil, as a ground of decomposition, is filled in by cadaveric fluid leaking out of natural orifices and violated skin (Vass, 2001; Dent et al., 2004; Statheropoulos et al., 2011). Moreover, various volatile compounds are released during about 10 days of this phase known as ‘black putrefaction’ (blackout skin) (Statheropoulos et al., 2011). Depending upon climatic conditions stages may also be identified notably saponification (in warm and moist environments) and mummification (dry heat, low humidity) after the onset of putrefaction (Vass, 2012; Ioan et al., 2017; Kori, 2018; Javan et al., 2019; Moraleda et al., 2022; Titus et al., 2022). While maggots pupate, the stage named “Advanced Decay” has its onset (around the 29th-day *postmortem*). The duration of the later stage reaches the 51st day of decomposition. Meanwhile, the lateral extent of the Cadaver Decomposition Island (CDI) can be expected. The CDIs stand for a creation of a greatly concentrated island of fertility (by carbon, nutrients such as phosphorus, potassium, calcium, magnesium, and nitrogen) as the outcome of microbial labor surrounded by floral and faunal accumulation in the soil beneath the cadaver (Carter et al., 2007; Clases et al., 2021). Between the third and sixth-month *postmortem*, butyric fermentation takes place, attracting insects that further feast on the putrid tissue (Carter et al., 2007; Statheropoulos et al., 2011). Eventually, only the skeleton, skin, and hair remain, as most of the soft tissue is disposed of (Ioan et al., 2017; Javan et al., 2019; Clases et al., 2021). “Skeletonization” also referred to as ‘Dry’ winds up the whole decay case (lasts from the 52nd to the 207th-day *postmortem*). Essentially during this phase, the organic and inorganic elements of the bones undergo natural diagenesis, which involves alterations in their proportions. It is postulated that “increased plant growth around the edge of the CDI” can be a key indicator of the “Dry” phase. Nevertheless, one proven fact is that elapsing hundreds of years is necessary to document the complete disappearance of skeletal remains (Vass, 2001; Carter et al., 2007; Perrault et al., 2015a,b,c; Ioan et al., 2017). A summary of the chemical compounds specific to each stage of decomposition is presented in Figure 1.

## 3. Volatile organic compounds in forensics

Volatile organic compounds (VOCs) are entitled to an individual approach. Their differentiation via the headspace analysis can contribute to confirm the human decomposition stages. Each stage, from early to advanced decay, has its own odor signature (Clases et al., 2021). Defining a cut-off point for individual VOCs is extremely challenging because volatile chemicals mutually overlap instead of creating seamless chronological chains. An alternative approach is to categorize VOCs by dividing them into three phases of decomposition: early (0–4 years), mid (5–20 years), and late (20 years +) (Vass, 2012). Anticipating the foregoing considerations, it is crucial to acknowledge the potential sources of the odor at the time of autopsy. According to Katherine C. Titus “*the decomposing body itself, biological fluids/tissues, internal cadaveric region, microbes, entomological specimens, and soil*” belong to the profile of *odor mortis* (Deo et al., 2020; Titus et al., 2022).

It is almost unattainable to indicate certain chemicals at a particular time during the decomposition process without any doubts. Several taphonomic factors make a significant contribution to altering the pattern of chemical production. Supplementary Table S1 specifies the most frequent and investigated volatile compounds based on available data. The essence of this section is to match the fragrance substance to the estimated time since death. Indeed, Burial Accumulated Degree Days (BADD) provides precise measurements that are useful in establishing a timeframe for compounds. In fact, BADDs are related to environmental conditions (temperature, humidity) prevailing in the research ambiance. Regardless, one of the proper assumptions is to gather and unify this and other general data as a current state of knowledge, afterward enriching them with novel findings (Vass et al., 2008). Basically, speaking of the scent of death a distinction is made between VOCs perceptible for humans and only for scavengers or cadaver-detection dogs. Further research is required to establish the specific target of the cadaver dog’s olfactory system. A great number of scientists hesitate among one, few, or whole profiles of VOCs as being responsible for attracting canines. Potential biomarkers of human decomposition include polysulphides such as dimethyl disulfide and dimethyl trisulfide as charged with a strong odor (Armstrong et al., 2016; Clases et al., 2021). On the other hand, nitrogen-containing compounds, i.e., putrescine (1,4- butane diamine) and cadaverine (1,5-pentane diamine) are treated to be a key for recognition via VR canines olfactory of human cadaver so far (Hoffman et al., 2009). However, in view of putrescine and cadaverine functionalities in forensic medicine and forensic science as training aids, the bottom line is characteristics of odor – cadaverine was specified by putrid and decaying flesh odor and putrescine has putrid and nauseating odor. Other substances with distinctive scent impressions (fecal, nauseating, fish-like odor), which have not been determined in explored literature as human-specific VOCs, are named indole, skatole, and pyridine (Statheropoulos et al., 2005a,b, 2007; Dekeirsschieter et al., 2009; Hoffman et al., 2009; Rosier et al., 2015).

The selection of volatile chemical compounds and their characteristics, as well as the influence of individual factors on their release, are summarized in Supplementary Table S1. The “decomposition phase” section refers to the phase of detected. The “approximate PMI” section indicates the extent to which PMI compounds were detectable in previous studies.

## 4. Sources of material for research on VOCs

Using human cadavers for the examination of VOCs, apart from body donation programs, is not only a logistically challenging endeavor restricted by ethical and legal considerations, but also would result in a complete profile of the scent of human cadavers. The solution to this problem is using human analog models, especially domestic pig carcasses (*Sus scrofa domesticus L*.) to cope with aforesaid difficulties (Dekeirsschieter et al., 2009; Stefanuto et al., 2015; Titus et al., 2022). The exploitation of surrogate human models in the guise of pig corpses is justifiable due to plenty of similarities among these species. These encompass factors: weight, fat-to-muscle ratio, internal anatomy (e.g., size of the chest cavity) and skin analogy, biochemistry, body composition, skin coverage with hair, gut fauna (owing to a shared omnivorous diet), immunological system, gross processes of decay (Matuszewski et al., 2020). Ethical issues are usually not used to limit most investigations because of conventional domestic pig breeding in nutritional destiny farms, which is connected with the frequent acquisition of pig carcasses from a licensed abattoir and less public sensationalization (Perrault et al., 2015a,b,c). Ventures relating to multiple donations of human corpses for scientific purposes are unforeseeable and unscheduled. Consequently, pigs are predominant over humans from the standpoint of the larger number of research models accompanied by a lower financial contribution (Dekeirsschieter et al., 2009; Rosier et al., 2015; Miles et al., 2020). Also, it is revealed that pigs (at least 30 kg weight) and human cadavers have insect fauna that is superimposed on each other. Unquestionably, porcine remains are successfully useful during local carrion-arthropod faunas’ investigation (Matuszewski et al., 2020; Miles et al., 2020). On the other hand, according to several studies, the divergence between pig and human cadavers’ decomposition and odor profiles was apparent (Schoenly et al., 2006; Brasseur et al., 2012; Stefanuto et al., 2015; Matuszewski et al., 2020). Pigs typically undergo more advanced stages of decomposition within the shorter period after death, leading to potential discrepancies. To substantiate this hypothesis, it was observed that active decay in human cadavers commenced between the 14th and 21st experimental days compared to pig carcasses, which reached the same stage in only 6 to 8 days during the summer trial (Knobel et al., 2019). Continued changes in mummification progress slower in pig remain. Moreover, in pig corpses noticeable is the phenomenon of rupturing the intestine and abdominal wall, observed in approximately 60% of pig remains during the bloating phase of decay. Such an event did not occur at all in human corpses. The cause of this difference is an exponentially larger intestine surface area for the activity of gut microbiota and, consequently, the production of large amounts of putrefactive gasses (Connor et al., 2018; Miles et al., 2020). Another mismatch applies to insect activity. Entomological occupation covers the entire pig carcass which leads to rapid soft tissue loss. The human head and groin are primarily inhibited by insects while the rest of the decaying body becomes mummified (Dekeirsschieter et al., 2009; Matuszewski et al., 2020). Comparable environmental conditions should appear during further studies in order to obtain a reliable comparison between the set of volatile compounds released from pig and human cadavers, at the specific time since death. In the meantime, research concerning the decomposition of pig carcasses may enable victim recovery canines not to be mistaken in differentiation between human and animal remains and improve the understanding process of decay (Vass, 2012; Knobel et al., 2019). The milestone for experimental forensic entomology and taphonomy research could be identifying potential human decomposition chemical volatile markers based on pigs’ investigation. The investigation in Australian temperate points out a group of volatile compounds specific to human remains i.g.: 3-methyl butyl pentanoate, 3-methyl butyl 3- methyl butanoate, 3-methyl butyl 2-methyl butyrate, butyl pentanoate, and propyl hexanoate as possible distinctive markers of human decomposition. Also, phenylethene and methyl benzoate correspond to them. Some VOCs are more attributed to pig remains and human one’s either. Those can be categorized as diethyl disulfide, methyl(methylthio)ethyl disulfide, 3-methylthio-1-propanol, pyridine, ethyl propionate, propyl propionate, propyl butyrate, and ethyl pentanoate (Vass, 2012; Rosier et al., 2015). Disappointingly, preceding results have been obtained by lab-controlled studies, therefore their implementation into practice is not warranted. Also, one of the scientific papers devoted to equating human and pig decomposition rates and odor profiles in the light of ambient weathering provides evidence that in winter months VOCs collected from both humans and pigs are more equivalent than during the summer months (Dekeirsschieter et al., 2009; Rosier et al., 2015; Perrault et al., 2015a,b,c; Forbes, 2017; Knobel et al., 2019). Pursuant to Blanar and Prada-Tiedemann's (2020) conclusions indole could be a possible odor biomarker of decay stages from bloating to active as well as dimethyl disulfide and dimethyl trisulfide might perform this function at the initial stages of decomposition but these findings applied to pig cadavers investigations. To summarize, pig cadavers can yield the very same VOCs released from human remains, although the abundance and appearance time constitute alteration in the whole terminal odor profile. Authors of diverse scientific papers have emphasized the reliability of findings only with regard to the early decomposition of human and pig cadavers because of differences in decay ratio (Dekeirsschieter et al., 2009; Stefanuto et al., 2015). In the review, the general profile of volatile compounds has been shown to be similar. As a result, pigs have offered comprehensive experimental baseline data in the field of decomposition, entomology, ecology, or taphonomy. Additionally, they determine acceptable odor composition, which supports contemporary as well as future explorations of potential human decomposition biomarkers (Dekeirsschieter et al., 2009; Vass, 2012; Rosier et al., 2015; Matuszewski et al., 2020). Cadaver-detection dogs could be trained using the scent of pig carcasses but further verifying findings are needed to support this. Pigs afford to gain an overall trend of human decomposition phenomenon. Moreover, the scientific community is unanimous that human carcasses are recommended for more accurately determining the time of death and only the usage of humans can highlight the direct human body decomposition model. The arising of human decomposition research facilities across the globe succors to this aim (Brasseur et al., 2012; Rosier et al., 2015; Armstrong et al., 2016; Knobel et al., 2019; Matuszewski et al., 2020).

The first world outdoor human taphonomic facility has been created in Tennessee. Also, they can be colloquially referred to as “body farms” considering the crime fiction novels’ prevalence. Subsequently, research establishments have been created in other locations in the USA (at least 8 institutions), in Australia, Canada, and the Netherlands (Williams et al., 2019; Doro et al., 2022) although taphonomic research with the exploitation of donated human corpses is not allowed in many countries. In each country that does not include legal and ethical restrictions, forensic facilities should be bound by the agreements when forensic facilities obtained the approval for development, the ethical agreements were hedging the body of donor or his family. An impeccable reflection of the definition, destination, and whole concept of body farms is the presentation of them as outdoor forensic anthropology research laboratories where donated human bodies during decomposition can be monitored and investigated in controlled conditions regarding several environmental and taphonomic factors (Baryah et al., 2019; Williams et al., 2019). They are intended to profoundly study *postmortem* human decay, ascertain the biological profile of the deceased and compare it with animal decomposition models. Results that have been revealed up to now, provided evidence of differences between porcine and humans mentioned earlier. Anthropology centers conducting studies uptake human cadavers apply to PMI estimation for death investigations, upgrading current knowledge, search, recovery methods, and identification of victim debris. Also, body farms play a key role in conducting training for law enforcement departments (Forbes, 2017; Baryah et al., 2019; Miles et al., 2020). Despite the undeniable fact that human taphonomic centers provide valuable data regarding the above-mentioned issues, the results obtained should not be extrapolated on a global scale. An argument in favor of limiting the use of data is the climate variability depending on the location of the facilities, and as it is widely known, environmental conditions crucially impact the decomposition of corpses. The establishment of Human Taphonomy Facilities in new locations is justified, among others, due to the continuous need to improve the methodology of locating and identifying victims of natural disasters, catastrophes, and terrorist incidents, or for the use of law enforcement agencies. Finally, according to the “National Commission for the Protection of Human Subjects of Biomedical and Behavioral Research 1978”, the use of human remains for research is limited by fundamental rights which are: “respect for persons,” “do not harm,” and “justice” (Williams et al., 2019; Miles et al., 2020).

## 5. Factors determining the VOCs profile

The factors associated with the generation of a specific profile of VOCs can be divided into two distinct categories. Extrinsic factors include time, temperature, humidity, oxygen, pressure, various types of soils, water environment, pH, and insect activity. Intrinsic variables include body mass, clothing, cause of death, toxicology, and various organs of the human body. These issues are closely related to practical aspects of the criminology department as the mentioned variables have a significant impact on the odor detected by HRD dogs. The scent of death is one of these characteristics and, if properly identified, can enable the choice of the most promising indicators of human presence and substantially reduce the false positive rate during the practical chemical analysis (Agapiou et al., 2015).

### 5.1. Temperature

Temperature is, along with the time variable, one of the most significant factors. Since it is always a constant variable, it cannot be omitted. Typically, the temperature is considered in research involving the emission of volatile organic compounds from decaying bodies, but it is not always accounted for in the results and discussion. In a study conducted at an average outdoor temperature of 24.1 degrees Celsius, which is very close to room temperature, nearly all chemical classes were detected, including aromatics (25 compounds), nitrogen (19 compounds), aliphatics (172 compounds), ketones (17 compounds), alcohols (12 compounds), esters (13 compounds), aldehydes (11 compounds), miscellaneous (8 compounds), acids (2 compounds) and sulfur (9 compounds) (Agapiou et al., 2015). Not all chemical classes are influenced by temperature (Vass et al., 2004) determined that the temperature had no effect on appearing of cyclic hydrocarbons, oxygen compounds, acids/esters, halogen compounds, and sulfur compounds. Noncyclic hydrocarbons are the only class impacted by temperature. In addition, it is uncertain if temperature influences nitrogen compounds (Vass et al., 2004). The most accurate studies on temperature as a variable are those that compare summer and winter VOC emissions. Forbes et al. (2014a) have carried out research comparing the summer and winter VOC profiles of pig carcasses in a climate of mid-latitude. During the winter period, several 116 VOCs related to decomposition were found, whereas in the summer period, 256 VOCs were discovered. Furthermore, no VOCs were found almost exclusively during the winter study since all chemical compounds were identified at some point during the summer period study. Additionally, numerous VOCs were discovered only in the summer trial for example (2,4-dithiapentane, 1-butanamine, 3-methyl-N-(3-methylbutylidene)-, indole, pyridine, 2-n-butyl furan, 2-propenoic acid, methyl ester, 3-methyl-2-butanol, 1-penten-3-one, 2-butenal, 2-ethylacrolein, formaldehyde, propanal). Moreover, at elevated temperatures, Rajamäki et al. (2006), detected a growing amount of dimethyl disulfide inside the atmosphere of chicken carcasses, which may have originated from methanethiol, supporting that some chemical compounds might not be detectable at lower temperatures (Rajamäki et al., 2006). A few years later, Knobel et al. (2019) conducted an analogous study comparing seasons and volatile characteristics in the Australian climate. Additionally, they aimed to compare VOCs unique to pig and human remains. Compared to the previous study, fewer VOCs were discovered overall. During a summer sampling, 77 volatile organic compounds were considered important, distinguishing the sampling from the control trial, with 74 VOCs assumed to lead out from the decomposition process. Throughout the winter sampling, several 29 volatile organic compounds were regarded as important, although 28 of these were presumed to emerge from the decomposition process. Furthermore, they determined in their study that neither the summer nor winter trial revealed any human-specific volatile organic compounds (Knobel et al., 2019). Overall, both kinds of research have reached the same result that there are more VOCs prevalent throughout the summer period. This is the result of the greater daily mean temperature, which has hastened the decomposition process rate and enhanced insect activity (Forbes et al., 2014a; Perrault et al., 2015a,b,c). In a study where the temperature was increased, a correlation was also shown between an increase in insect activity and the emission of more VOCs by decaying corpses. The presence of many bodies in one place in one experiment as opposed to a single body in the other trial caused an increase in temperature (Ueland et al., 2021). Statheropoulos et al. (2005a,b) sought to investigate the volatile profile of decomposing human bodies. In their experimental design, two human cadavers were utilized. During sampling, the average ambient temperature reported for the first cadaver was 13.75 degrees Celsius, and for the second cadaver, it was 17.45 degrees Celsius. Even though the scientists did not assess the differences between cadavers, the result indicates a small increase in VOC concentration in practically every chemical class observed. This validates the findings and the positive correlation between temperature and the concentration of volatile organic compounds emitted by decaying remains (Statheropoulos et al., 2005a,b).

### 5.2. Type of soil

Noteworthy is the distinction between VOCs released by bodies buried below ground. Nineteen of the thirty crucial markers of human decomposition compounds reported in a burial scenario were also identified when gathering air samples above decaying carcasses on the surface, acknowledging the assumption that they naturally occur from the corpse. Predicted compounds that have not been observed during the ground decay may imply that these components are produced during strictly anaerobic mechanisms or necessitate the conversion of a base compound by microbial activity (Vass et al., 2008). Other studies reached the same conclusion, but more volatile organic compounds were detected: 119 in the surface soil, 72 in the grave headspace before probing, and 83 after probing (Forbes et al., 2016). That further implies that probing technique is an important factor. However it only applies in an experimental setting. In general, the air data points above the burial site contained fewer compounds than the samples collected below the remains. As previously mentioned, this may have been caused by anaerobic processes in the soil and microbial activity, as well as the rapid dispersion of volatile organic compounds throughout the air as a result of wind, evaporation, and other environmental characteristics (Forbes and Perrault, 2014). Furthermore, it is possible that the volatile profile would vary depending on the soil type at each location where a body has been discovered. Clay soil is an example of this. Several enzymes and antimicrobials, for instance, are known to be absorbed by it. It is probable for microbial enzymes or perhaps even decomposition products to bind to clay particles and become rendered inactive. In addition, the reduced permeability of the condensed clay soil may inhibit any odors emanating from a dead body. This may hinder compositional odor (Turner and Wiltshire, 1999). Alternatively, distinct types of soil and their resident microbiological populations may not affect the appearance, release, or identification of these substances (Vass et al., 2008). It is not just the presence of a variety of soil types but also a range of soil moisture and pH levels that are encountered frequently. In most instances, rainfall contributes to most soil environments. On days with heavy precipitations at experimental sites, more compounds were detected than on days with lighter rainfall (Perrault et al., 2015a,b,c). Water serves as a solvent for polar volatile organic molecules when the soil is wet but as a competitor for the adsorbent surface on the soil surface when it is dry (Forbes and Perrault, 2014). Different study that considered precipitation a possible factor in VOC release, found that, it in fact, influence VOC, mostly noticeably by reducing decomposition VOC abundances (Forbes et al., 2016). Acidity and alkalinity are two additional variables that influence cellular biochemical processes and the catalytic activity of enzymes. Anaerobic burials are typically acidic as a result of microbial fermentation and the release of organic acids, while proteolysis creates alkaline ecosystems (Vass, 2011). Across one day of laboratory activity, soil acidity, and higher moisture likely influenced VOC preservation, resulting in a rise in the number of molecules detected (Perrault et al., 2015a,b,c). Consequently, more VOC’s are being detected in moist and acidic soil. Important considerations also include the burial’s depth. The grave level has a considerable impact on the creation of various chemical classes, most probably due to the higher partial pressure of oxygen in the relatively shallow pit, where it affects microbial activity as well as the emergence of compounds (Vass et al., 2004). Consequently, fewer substances are released from the deeper burial site.

### 5.3. Water environment

Since an aquatic ecosystem is very different from a terrestrial environment, differences between them will also have an impact on volatile compounds that emerged from human corpses. Based on a study comparing VOC emissions from surface-deposited versus submerged pig remains it can be concluded that there are significant differences. The volatile profile of the surface deposited was distinct from that of submerged pigs. Overall, in the research study, 74 volatile chemicals were identified. The submerged trial contained 41 volatile organic compounds, while the surface deposited one contained 70 VOCs. Regarding both of the trials, esters constituted a minority chemical class. They were only identified in one case. This may have been because of the greater mass of remains or the increased temperature to which they were subjected. Fever chemical classes have been detected in the submerged site compared to this on the surface. There was no evidence of cyclic hydrocarbons or ethers in the submerged pig’s trial. Butanal was the only aldehyde identified in the submerged trial, whereas no aldehydes were detected in the surface-deposited one (Irish et al., 2019). Based on this information, it is possible to conclude that butanal is a VOC unique to water. Furthermore, based on the gas–liquid chromatography performed by different authors, it was discovered that the carcasses which had been submerged in water for a certain period contained a wide range of alcohols, sulfur compounds, and amines (Tomita, 1975).

### 5.4. A type of biotope

Not only underground or in the air, but bodies of the deceased can be discovered anywhere. In many of these instances, human remains have been discovered following natural disasters. People discovered in collapsed structures are one such case. In addition, the biogeoclimatic zone, as well as the urban environment can influence VOCs. Agapiou et al. (2015) carried out a field experiment in stimulated collapsed construction conditions in a pig model. One carcass was buried in a cement tunnel with an open bag, while the other was buried in a tunnel with adequate soil to completely cover the body. As a result of their investigation, they have concluded that there are notable differences between the chemical profiles at the two locations used for sampling. Particularly, sulfur compounds were the most prevalent chemical components on the first day at the sampling point with an open bag. On the third day, the concentration of aliphatics, followed by nitrogen, aldehydes, and sulfur compounds, persisted. On the following day, aliphatic and aldehyde concentrations increased, as well as aromatic and sulfur compounds. Aromatic compounds were the group with the highest concentration on day five. In turn, on the first day, no significant observations were made at the sampling point with soil covering the body. Aliphatics, alcohols and ketones were prevalent on the second day. The concentration of sulfur compounds, aliphatics, aldehydes, and ketones increased on the third day. The most significant substances detected on the following day were alcohols, acids, sulfur and nitrogen compounds. On the fifth day, the most abundant classes were both types of hydrocarbons. As previously stated, anaerobic reactions are preferred when corpses decompose in the soil. This varies in an open space of a concrete tunnel, resulting in a different emission of volatile organic compounds. In addition, underground VOCs diffuse into the soil, where their chemistry is altered (Agapiou et al., 2015). The same type of study was carried out by Ueland et al. (2021) with human corpses instead of pig ones. In a prior study, sites in and out of concrete tunnels were compared, however in this trial, the volatile profile relates to the two distinct VOC collection centers in the simulated disasters area. These trials varied as bodies were either arranged singly or in groups. Furthermore, in the first trial group, the bodies were covered with bricks and concrete, whereas in the second group, that was not the case. Esters, aromatics, aldehydes, ketones, alcohols, hydrocarbons as well as sulfur, phosphorus, and halogenated compounds were more abundant in the second trial, which lacked concrete and brick coverage and in which bodies were stacked closer and more together. In addition, more precipitation and a slightly lower temperature were observed in the second trial, which could also have affected the results. In general, it was observed that the decomposition process and VOC release were affected by the site of the corpses in the simulated disaster area (Ueland et al., 2021). Human remains are often discovered for reasons other than natural occurrences. The open space environment, such as a forest or crop field, and the urban site must also be taken into consideration. In an experiment where pig carcasses were placed in three aforementioned distinct biotopes, numerous differences in volatile profile were identified. The three selected sites shared a core of 35 volatile organic compounds. As the corpse’s odor signature varies in each of those biotopes, many compounds were not detected, most likely due to the character of each environment surrounding each carcass which interacted with VOC production. The rates of decomposition differed significantly among those sites. The distinction between open-air places and the urban biotope is the most significant. Numerous contaminants were sampled in the urban biotope, resulting in significant background noise. Such compounds may disguise the existence of cadaveric volatile organic compounds (Dekeirsschieter et al., 2009).

## 6. VOC profile of tissues and organs

Despite the fact that the discovery of entire human corpses would be advantageous, each of these can generate a volatile profile that is distinct or comparable to that of the entire corpse. Every tissue is composed of various cells and chemical compounds that undergo diverse chemical reactions under varying environmental factors. Each of these tissues can produce its unique volatile profile during decomposition. Moreover, in many instances, only body parts such as organs, blood, urine, or even residual air are discovered at various sites.

A study compared the volatile organic compounds released by the decaying organs of chickens, pigs, and cows. Fresh and decaying bones can emit hexane, toluene, nonanal, and p-xylene among other compounds. While all these compounds are present, alcohols, aromatic hydrocarbons alkanes, and aldehydes predominate. The samples of decomposed animal fat constituted more aldehydes compared to any other chemical class, most likely because of the breakdown of fatty acids. During the decay process, sulfides present in the skin induce desulfhydration to generate sulfur-containing molecules including thiols and dimethyl disulfide, which was found in analyzed cow and chicken skin samples. Muscle proteins require more time to decompose than epithelial tissue ones due to the higher stability of their molecules. Indole can be perceived as a breakdown byproduct of skatole, and skatole itself can be the product of proteolysis and was discovered solely in muscle samples from cows and pigs, whereas skatole was not detected. Alkanes and aromatic hydrocarbons were abundant in both fresh and degraded muscle samples. Some acid esters/acids were found, depending on the stage of decomposition (Cablk et al., 2012).

Blood as a material for VOC analysis is also significant as its odors closely resemble those of human decomposition (Ruffell and Powell, 2021). In a study comparing the volatile profiles of fresh and degraded blood, researchers determined that each generates profoundly different volatile organic compounds, that change over time. The primary components of freshly drawn blood were ketones and alcohol. Throughout the study, 1-octen-3-ol remained the most prominent component of the volatile blood profile, despite the increased abundance of aldehydes, ketones, and alcohols in degraded blood. Even at the time of collection, measurements taken at room temperature contained a greater concentration of volatile organic compounds, than under refrigeration and in freezing conditions. Freezer-stored and refrigerated samples showed a wide range of VOC variability. The blood samples kept for 1,3 and 5 weeks showed the presence of 2-heptanone, 1-octen-3-ol, and 2,5-octanedione (Forbes et al., 2014b).

Residual air is another factor that must be considered. An analysis of 63 volatile organic compounds in expired air revealed the presence of ketones, alcohols, hydrocarbons, esters, acids, aldehydes aromatics, chlorinated hydrocarbons, and heterocyclic compounds. 38 and 46 substances have been identified, respectively, in urine and blood. They exhibit the same chemical diversity as expired air, containing ketones, alcohols, hydrocarbons, sulfides, aldehydes, and heterocyclic compounds. Acetone, butane 2,2,3,3, tetramethyl, and toluene were the only substances noted in all samples, while phenol and isoprene are more likely to be present (Statheropoulos et al., 2005a,b). The significance of considering not only body parts but also remnant air is demonstrated by this study. Human remains and residual parts of a body have been found on a wide variety of surfaces, thus this is another important factor to consider. Surfaces such as glass and metal rapidly emit the relatively modest quantity of scents they have absorbed into the surrounding air. Since a given mass of plastic material may comprise much more scent particles than an equal amount of air can absorb in equilibrium, plastic that has had sufficient time to reach equilibrium with the original smell origin will release odors at a relatively high rate and over a relatively long period of time (Goss, 2019). Rust et al. intended to examine the chemical odor profile of volatile organic compounds generated by fresh and aged human blood on different surfaces. In their investigation, an assessment of chemicals by given material reveals that no specific chemical class leads the profile toward the porous surface being cotton or non-porous surface being aluminum and that, a comparable degrading mechanism is indeed occurring for blood placed on any material. Based on their respective density, aromatics, and aldehydes favored the volatile profile of non-porous measurements, but this was not the case for porous samples, where alcohols, aromatics, and hydrocarbons dominated. In addition, they pointed out that after 12 months, the volatile profile cannot differentiate between porous and solid surfaces. Furthermore, a subsequent study by the same authors conducted a year later compared blood samples deposited on weathered wood and concrete surfaces from the outdoor area. In this instance, environmental factors including the weather were taken into consideration. During the trials after 24 h, the volatile profile of blood-saturated and control concrete trials had similar chemical compositions, with a large percentage of the profile comprising heptanal, nonanal, and 2-pentylfuran. Blood surface profile could be differentiated from the control for up to 2 months of weathering but was no longer discernible after almost 3 months of weathering. Similarly, weathered wood sample trials were also distinguishable from the control for up to 2 months after exposure. Both trials were influenced by the weather differently, for example, varying amounts of blood on wooden surfaces were lost due to the surface drying and peeling caused by wind and high levels of rainfall (Chilcote et al., 2018).

## 7. Toxicological analysis of VOCs

The odor of decomposing human body is not always caused by the emission of volatile organic compounds. Toxicology is one of the aforementioned intrinsic factors, such as the volatile profile of various body organs. One of the most important clinical aspects is that this type of odor can be recognized by the human olfactory system in addition to chemical methods of recognizing VOCs. Ohkubo et al. (2020) presented a case report on a 60-year-old man whose body was found 300 meters from his residence. The death scene reeked like sulfur. Hydrogen sulfide was identified in the deceased’s oral cavity, as well as the nasal and oral cavities smelled specifically upon autopsy. During the autopsy, hydrogen sulfide intoxication was found as the cause of death. In addition, qualitative tests for hydrogen sulfide were positive (Ohkubo et al., 2020). On the other hand, during a ten-year period, 17 cases of cyanide poisoning were documented in the city of New York. In this instance, the almond-bitter odor and pink lividity make for suspecting cyanide poisoning (Gill et al., 2004). Another case describes a man in his fifties who was discovered dead at his son’s residence, along with a small towel smelling of naphthalene. The toxicological evaluations showed the existence of TFP and its metabolite in the *postmortem* test, along with methyl naphthalenes and naphthalene, using liquid chromatography-mass spectrometry and gas chromatography (Hikiji et al., 2013).

## 8. The character of odor mortis depends on thanatomicrobiome

The thanatomicrobiome (*−thanatos*, Greek for death) is the study of microbes colonizing internal organs after death (Javan et al., 2016). Temporal change of the VOC profile may be an important indicator for determining PMI, where it is significant to analyze VOCs as partly by-products of microbes’ activity. After death, bacteria belonging to the natural physiological flora use human corpses as a perfect medium, enabling their growth. During the “bloated stage,” the intestinal and other anaerobic bacteria begin to digest tissues. Their metabolic processes are responsible for producing gasses. It results in creating internal pressure, which leads to the oozing of fluids from the natural orifices of the body and a strong smell of ammonia (Goff, 2009; Wójcik et al., 2021).

At the beginning of the decay at time 0, the major factors responsible for the formation of VOCs were found to be *Moraxellaceae, Aerococcaceae*, and *Clostridiaceae*, at 48 h *Enterobacteriaceae* and to a lesser extent *Leuconostocaceae*, while at 96 h *Flavobacteriaceae, Xanthomonadaceae, Enterococcaceae, Erysipelotrichaceae, Porphyromonadaceae, Tissierellaceae*, and the unclassified *Gammaproteobacteria* group. *Erysipelotrichaceae, Xanthomonadaceae*, and especially *Tissierellaceae* significantly contributed to indole and phenol emissions. However, *Xanthomonadaceae* was the only taxon especially correlated with the presence of phenol. The families *Tissierellaceae, Erysipelotrichaceae, Xanthomonadaceae,* and *Enterobacteriaceae* were responsible to the greatest extent for the appearance of acetic acid, with *Tissierellaceae* and *Enterobacteriaceae* representing the most influential taxa. The temporal dynamics of hexanoic acid were most significantly associated with the taxon *Moraxellaceae.* Overall, it can be concluded that the presence and time dynamics of the *postmortem* VOCs presented are not stochastic but significantly correlated with bacterial taxa (Pascual et al., 2017). In the later study by Cernosek et al. (2020) three *postmortem* bacterial isolates: *Bacillus subtilis, Ignatzschineria indica*, and *Ignatzschineria ureiclastica,* were cultured and monitored over 5 days for their VOC profile. Each of the three species showed an individual VOC profile including typical decomposition VOCs, which changed over time. *Bacillus subtilis* was found to be responsible to produce compounds such as 1-butanol, 2-pentanone, and 3-methyl-2-pentanone, which increased steadily over time, and 1-methylcyclohexene, 1,3-pentadiene, and butyl ester acetic acid, which increased rapidly and then decreased over subsequent days of monitoring. Additionally, 1-butanol was the VOC produced by these bacteria in the largest concentration. *Bacillus subtilis* is a facultative anaerobe, so the presence of oxygen may lead to a change in metabolic pathways and secretion of 1-butanol, the amount of which increases with the growth of the population of this bacterium (Schotsmans and Márquez-Grant, 2017; Cernosek et al., 2020). In a previous study by Lee et al. (2012), only two of the above-mentioned compounds: 1-butanol and acetic acid methyl ester were identified as chemicals produced by *Bacillus subtilis* (Lee et al., 2012). Dimethyl disulfide and 2-butanone also were found as products of this bacteria; however, these two VOCs were found to be insignificant in the study by Cernosek et al. (2020).

*Ignatzschineria indica* produced the largest amount of VOC among the observed bacteria, it was as many as 15 different VOCs. The amount of dimethyl disulfide increased notably during the monitoring period and was significantly higher than all other VOCs produced by the rest of the bacterial species monitored, and at the peak of its production, its amount was 2 times higher compared to all other VOCs formed during decomposition in this study. Thus, this species of bacteria may be responsible for the production of the large amounts of dimethyl disulfide detected in the early stages of decomposition (Cernosek et al., 2020). *Ignatzschineria ureiclastica* produced 2 compounds: 3-methyl-2-pentanone and phenol, however, along with the phenol, it made increasing amounts of benzaldehyde. Phenol, characteristic of both species of *Ignatzschineria,* is an antiseptic chemical that inhibits the growth of other microorganisms. Benzaldehyde and its derivatives also exhibit antibacterial properties. This may represent a competitive strategy for the decomposition of this bacterial family (Pascual et al., 2017) also observed phenol as a compound produced by the *Xanthomonadaceae* family, whose representative is the genus *Ignatzschineria* (Pascual et al., 2017).

The results of these studies indicate that each microbial species produces a different VOC profile, which includes compounds commonly noted in decomposition smell studies. Interactions between species and sources of nutrients may also play a role in the dynamics of VOCs released from *postmortem* microbial activity. In addition, it is crucial to realize the nonlinear nature of microbial VOC production (Cernosek et al., 2020). Cappas et al. (2022) concluded variations in VOC emissions are dependent on sampling location because communities evolved differently depending on it. The *Moraxellaceae, Planococcaceae, Lactobacillaceae*, and *Staphylococcaceae* families were most responsible for these changes. The nostrils were identified as the most suitable predictor of the decomposition stage based on bacterial activity. Additionally, *Planococcaceae* and *Tissierellaceae* were especially associated with the presence of indole (Matuszewski, 2021). However, further research is needed to understand other *postmortem* bacteria colonization processes and their volatile emissions as this may facilitate the determination of the PMI.

## 9. Entomological aspects of the VOCs profile

The decomposing body serves numerous purposes for carrion insects, such as food, finding a mate, reproduction, oviposition, or offspring development (LeBlanc et al., 2009). Therefore, their survival depends on effectively locating in decaying organic matter, thus they rely more than other organisms on detecting volatile chemicals by olfaction to survive (Vickers, 2000). Insects’ succession patterns can help in the assessment of PMI (Villet and Amendt, 2011). In order to increase the efficiency of this estimation, it is crucial to recognize which VOCs draw insects to a cadaver (Yoho, 2009).

Studies showed that a wide range of forensically important species is attracted to dimethyl trisulfide and dimethyl disulfide. Yoho (2009) reported a significant response of *Phormia regina* to dimethyl trisulfide in a field study with pitfall traps. Dimethyl disulfide, likewise, lures this species into the traps, but in considerably smaller amounts. *Lucilia sericata* showed the most notable electroantennography (EAG) activity to dimethyl disulfide in experiments carried out by Frederickx et al. (2012a,b). On the other hand, Zito et al. (2014) did not register *L. sericata* antennal response to dimethyl disulfide (Zito et al., 2014). In another study, dimethyl disulfide occurred as unattractive to *L. sericata* too, as none were captured in dimethyl disulfide baited traps (Yoho, 2009). This discrepancy may be brought on by the fact that different concentrations of dimethyl disulfide were used in two trials. According to Dekeirsschieter et al. (2013), *Thanatophilus sinuatus fabricius* exposed to dimethyl disulfide and the control in a Y-tube olfactometer was mostly attracted to the investigated chemical compound, independently from its concentration or sex of a beetle. Depolarization of *T. sinuatus* antennae was most stimulated by dimethyl disulfide as well (Dekeirsschieter et al., 2013). On the other hand, Yan et al. (2018) found that dimethyl trisulfide presented to *Lucilia cuprina* elicited relevant reactions in behavior bioassays and EAG responses. The antenna of gravid *L. cuprina* was able to sense even as small an amount as 0.02 ng of dimethyl trisulfide (Yan et al., 2018). Nevertheless, it seems that EAG may not be an ideal method for determining which volatile compound will attract carrion insects in practice. For instance, in accordance with a study from Zito et al. (2014), *Musca domestica* responded to dimethyl trisulfide with antennal depolarization in EAG, whereas there was no behavioral response in the field experiments (Zito et al., 2014).

The single volatile chemical compound may be the attractant for carrion insects, but the synergism between two different compounds can have an even greater impact. The study from Trumbo and Dicapua (2021) indicated that the occurrence of carrion-frequenting beetles and total silphids captured rates were higher in the traps with dimethyl trisulfide addition compared to the control. However, dimethyl trisulfide coupled with methyl thiolacetate outperformed other tested forms of baits in relation to a number of lured beetles, with the exception of *Necrophila americana larvae*. Interestingly, larvae of *N. americana* were found to migrate between different decomposing remains, hence PMI assessment based on silphid larvae development would not be accurate.

Depending on the physiological state or sex, various VOCs may act as a repellent or an attractant for an arthropod. Males of *Dermestes frischii* present positive EAG responses for all decomposition stages, except for the fresh stage. Nevertheless, at the behavioral level, only active and advanced stages of decay drew them in. Females of *D. frischii* experience cadaveric VOCs differently though. They detected VOCs only from the advanced phase, but none of the decomposition stages elicited their behavioral reaction. These data support the idea that females only react to the cadaver odor when males occur on the remains and emanate pheromones that attract the opposite sex (Martin et al., 2020a,b). When evaluating the preference of *Nicrophorus vespilloides*, gravid beetles are attracted more to the fresh mouse carcass than the older carcass. What is intriguing, those female beetles spent more time with an empty control compared to the aroma from an old carcass, which may indicate that VOCs from older carcasses are received by gravid beetles as repellents (Delclos et al., 2020). On the other hand, young females of *N. vespilloides* without fully developed reproductive organs were found to favor more advanced decomposition stages (von Hoermann et al., 2013). Moreover, phenol might be a cue substance for immature *N. vespilloides* females since it occurs in high quantities during later decomposition stages and was an EAD-active compound when presented to those insects (von Hoermann et al., 2016). Additionally, it was demonstrated that indole was associated with repelling gravid females *L. sericata* from the cadaver but wasn’t affecting non-gravid females. Due to this, gravid females can avoid older remains for oviposition (Brodie et al., 2016).

Nowadays, more attention is being paid to the influence of carrion insects on odor mortis, because such modifications might affect the time and pattern of appearance of succeeding insects (Kasper et al., 2012). According to Kotzé et al. (2021), rat carcasses colonized with *Cochliomyia macellaria* abounded in acids and aldehydes. In contrast, much more sulfur- containing compounds were emitted from uncolonized remains. During the course of the ten-day collection period, the variety of volatile chemicals from colonized treatments stayed constant, while uncolonized carcasses presented a steady decline in the diversity of VOCs (Kotzé et al., 2021). However, in this study, the concentration of sulfur compounds emanated from chicken carcasses colonized by blowflies and uncolonized ones did not differ statistically (Recinos-Aguilar et al., 2019).

The topic that is currently getting noticed is that the insects themselves can serve as a sampling material for decomposition VOCs collection. Blanar and Prada-Tiedemann (2020) compared the composition of scent profiles emitted by *Calliphora vicina larvae* and tissue – each collected from a porcine corpse. Tissue samples yield 134 compounds, whereas maggot samples are 107 compounds, and 30 of them were specific to insects’ samples. Functional group proportions were very alike in the case of both matrices. It was postulated that maggot samples accurately depict the VOCs profile typically reported in cadaveric research since they emitted compounds like dimethyl disulfide, dimethyl trisulfide, phenol, and indole. Moreover, researchers tried to evaluate if larval mass samples emit specific VOCs pattern for particular decomposition phases. Dimethyl disulfide, phenol, indole, nonanone, dimethyl trisulfide, and undecanone were among the high-frequency compounds that showed characteristic fluctuations in quantity depending on the decomposition stage (Blanar and Prada-Tiedemann, 2020). Taking it into consideration, odor signatures from maggots may be useful in PMI estimation, especially when there is no corpse, and the only trace is entomological evidence (Blanar, 2019). Another approach is the assessment of flies’ age based on VOCs, which subsequently might help in the determination of PMI. In a study carried out by Frederickx et al. (2012a,b), larval stages and pupal stages of *Calliphora vicina* showed some differences in emitted compounds. All acidic substances (except for acetic acid), nitrogen compounds, and cyclic alcohols were released during larval development. On the contrary, sulfur compounds, ketones, alcohols, and aldehydes were specific for pupae. In addition, dimethyldisulphide was the only compound detectable throughout the whole pupal phase (Frederickx et al., 2012a,b).

## 10. The use of animals in the detection of VOCs in forensics

Dogs (*Canis lupus familiaris*), due to a combination of their unique abilities such as their olfactory system, the ability to cooperate with humans, being good at observing their surroundings, and willingly following orders, have been used by people in many areas. In forensic research, canines have worked since the 19th century as valuable tools (Nizio et al., 2017). Human Remains Detection (HRD) dogs, also called cadaver dogs have been trained to find human remains since the early 1970s (Grebenkemper et al., 2021) and nowadays they are one of the main methods in this field. Since then, multiple scientific studies have appeared.

Cadaver dogs can detect minimal amounts of specific human decay odor (Glavaš and Pintar, 2019). Studies showed that HRD dogs are not only highly sensitive (Furton and Myers, 2001) but they also characterize a high selectivity, being able to isolate and detect trace amounts of odors even in the presence of many other foreign scents such as those naturally present in the environment. The study by Alexander et al. (2015) proved that HRD dogs had 99.8 percent success in identifying grave soil samples in 711 trials and 96.8 percent accuracy in recognizing soil solutions from grave soils. With at least 75 percent accuracy canines identified grave soils to 915 days PMI. Furthermore, the soils where the bodies were removed after 667 days were recognized by the HRD dogs faultlessly (Alexander et al., 2015). Moreover, the cadaver dogs can achieve outstanding results in finding residual odor of human decomposition on textiles that are not associated with human remains with sensitivity (75–100), specificity (91–100), and a positive predictive value (90–100), negative predictive value (90–100) and accuracy (92–100) (Oesterhelweg et al., 2008). Although in the study by Chilcote et al. (2018) the GC × GC-TOFMS method proved to be more sensitive for the detection of blood, HRD dogs still achieved satisfactory results, because the blood which weathered for a week on concrete and a month on wood could be detectable by them (Chilcote et al., 2018). But on the other hand, in the study by Riezzo et al. (2014) dogs were found to be the most accurate tool for detecting low blood concentrations from human cadavers (Riezzo et al., 2014). In addition, HRD dogs can locate even individual human teeth in the field [100] and detect diluted decomposition fluid (Buis et al., 2015).

However, the dog breed is still an essential factor for some trainers, but on the other hand, greater emphasis is placed on temperament and the ability to collaborate with humans. From the results of a survey by Martin et al. (2020a,b), according to handlers’ responses, agility and stamina are also essential. However, the length of fur, size of the dog, or percentage of muscle mass is not considered necessary in selection (Martin et al., 2020a,b). Many studies recognize a dog’s motivation, high level of intelligence, and obedience, yet independent when working off-leash as a determinant of cadaver search success (La Toya et al., 2017). Generally, breeds of dogs that have been used in herding, guarding, hunting, and sports in the past, are selected (La Toya et al., 2017; Grebenkemper et al., 2021). The preferred species are Belgian Malinois (Malinois Shepherd), German Shepherd Dog (Grebenkemper et al., 2021), English Springer Spaniel, and Labrador Retriever (Rooney et al., 2007; Martin et al., 2020a,b). Golden Retrievers, Australian Shepherds, and Border Collies are also used (Page, 2008). It also depends on the country where the training takes place. In Russia, unique Jackal-dog hybrids were created for this purpose. In U.S. Bloodhounds are commonly used (Ferry et al., 2019). However, not every individual of the desired breed will be suitable, it may turn out that other breeds will be suited to investigative work (La Toya et al., 2017).

To be successful in searching, canines require proper training. An HRD dog team consists of one dog and one handler. It has been proven that the only factor that matters in HRD dog training is competent performance, which should be the major purpose of all dog handlers (Alexander et al., 2015). Locating the desired odor targets is the focus of most HRD odor training [94]. HRD dogs should be tested to determine their alarm responses to 30 compounds of VOC, individually and in combination, with proportions specified in the DOA (Decompositional Odor Analysis) database (Vass et al., 2008). The cadaver dog should be trained on various decomposition materials, including fresh, putrefied, and skeletonized material, to detect the complete range of odors (Cablk et al., 2012; DeGreeff et al., 2012). Due to the difficulties of access, storage, and ethical restrictions on the disposal of whole human remains, dog trainers must utilize various training aids (Cablk et al., 2012). Thus, training material can be natural like soil, various parts of the human body, e.g., placenta (Glausser, 2009), bones, blood, teeth, body fluids, muscle tissue, or substitutes created especially for this purpose (e.g., Pseudo™ Scents) (Nizio et al., 2017). Moreover, some artificial materials like Sigma Pseudo™ Corpse scent kit consist of corresponding to early decay – Sigma Pseudo™ Corpse Scent Formulation I and post-putrefactive decay – Sigma Pseudo™ Corpse Scent Formulation II (Stadler et al., 2012).

The study by DeGreeff et al. (2012) presented effectiveness utilize the sorbent material containing the odor as a training aid, which ensured a high percentage of correct dog responses close to 90, and a low rate of false positives (DeGreeff et al., 2012). In some countries, especially in Europe, domestic swine (*Sus scrofa*) is still used as a training aid (Cablk et al., 2012), due to their internal anatomy, fat distribution and the ratio of it to muscle, size of the thoracic cavity, body hair distribution, structure, and density in the form of lack of heavy fur, and omnivorous diet and a monogastric digestive system, which can result in intestinal microbiome similar to humans (Schoenly et al., 2006). HRD dogs should also have access to training aids specific to human remains at different stages of decomposition and to respond to different environmental conditions in which decomposition occurs due to significant differences in VOC emissions to optimize success in finding human remains in different circumstances of death (Caraballo, 2014; Irish et al., 2019). During training, dogs search for a target in a defined area which is called a “problem” and it can be “blind” or “known” In a blind problem the handler knows neither how many targets have been placed nor where the targets are located, whereas, in a known problem, the handler knows a number of targets and localization of them (Cablk and Sagebiel, 2011). HRD dogs may also be “cross-trained,” meaning they have been trained to more than one target scent, such as both alive human and corpse (Lit and Crawford, 2006). Canines are trained to give a clear signal to their handlers that they have found the target – human remains through a trained final response (TFR) also known as “the alert.” A “passive alert” includes inactive behaviors such as a sit or a down and an “aggressive alert” includes active behaviors such as scratching, barking, or digging (Cablk and Heaton, 2006). The handler is then able to interpret the signal as the definitive discovery of the specific human odor of decay. Some handlers use a probe during searches of remains in the soil to increase the number of VOCs available for detection. Then the ground is ventilated for half an hour and after that, the dogs can start searching the probed area. Unfortunately, this method is invasive because it may damage a corpse (Forbes et al., 2016). According to a survey 41 conducted in 11 countries, the HRD dog training process and scent lineup procedures used by the police in their countries are unstandardized and vary in many ways. The significant differences were in the degree of blindness required, the person who calls an alert (handler or experimenter), and the number of dogs with handlers that can work (one or more than one dog). However, the greatest similarity was noted between the use of control and zero trials, collection of decoy scents from individuals of similar gender and race as the suspect, materials for holding scent, frequency of cleaning and changing stations, and use and timing of rewards (Ferry et al., 2019). Moreover, many studies describe the best environmental conditions for odor detection as 20% or higher humidity and wind speeds of 8 km/h or more. The most optimal temperatures are between 4 and 16°C. However, some studies have shown that dogs can operate in sub-zero temperatures. When the temperature is too high, dogs begin to pant to cool down, which reduces their effectiveness in searching (Stadler, 2013).

Despite the huge potential of using dogs to detect human remains, there are still some difficulties and limitations to be faced. The most important difficulty is the availability of training aids. Natural odor sources, including materials such as human tissue removed at autopsy, decomposition liquid, or soil from a crime scene are difficult to obtain because of the law and may pose a biological risk to the dog and handler (Stadler et al., 2012). Whereas, while domestic pig carcasses are still the most commonly used training aid of animal origin, a study comparing human, chicken, pig, and cow carcasses found that chicken carcasses have 60% of the common VOCs with humans while pigs have only 23% which is seven of thirty human-specific compounds. In addition, pigs have 9 unique VOCs not found in humans (Rosier et al., 2016). Human remains are characterized by the production of more esters than animal remains, which is the most significant discrepancy between their VOCs from decomposition (DeGreeff et al., 2012). Furthermore, 30 of the VOCs have been identified as only specific to humans, therefore no animal is able to imitate human odor 100 % (Vass et al., 2008). In addition, the alternative, namely artificial training aids, often have an oversimplified chemical composition, that is also unable to reproduce the smell of decomposition (Nizio et al., 2017). Furthermore, the chemical composition of pseudo-scents is often unknown and unavailable and after testing one of them, it turned out that it does not contain any of the key VOCs (Stadler et al., 2012). Some synthetic aids included diamines: putrescine and cadaverine which were thought to be the main contributors to human decay odor in the past. However, research into the VOCs produced by the pig and human decomposition was unable to identify them, so their effectiveness as a training aid must be questioned (Stadler, 2013). On the other hand, there is a concern that using certain types of tissue as natural training aids may not match the smell of an intact human cadaver (Nizio et al., 2017). It is currently unknown which chemicals trigger a reaction in HRD dogs, although dogs may rely on several VOCs to identify carcasses. Therefore, this oversimplification of decomposition odor may result in poor odor recall during training.

The study by Prada and Furton (2018) describes the sense of smell in three species of birds: Homing Pigeons – domesticated rock pigeons (*Columba livia*), Turkey Vultures (*Cathartes aura*), Domestic Chicken (*Gallus gallus domesticus*), proved that birds can be a potential tool for odor detection in the future, as they have a well-developed olfactory apparatus and provide opportunities related to its use and can become an alternative to dogs. In birds, as seen in the example of the domestic pigeon, the ability to learn environmental odors in correlation with wind direction has been observed. In addition, the sense of smell plays a fundamental role in chemical signaling, intercommunication, and early experience in birds. However, further researches with a different perspective on birds as a viable and working method for forensic olfactory tasks are necessary (Prada and Furton, 2018).

## 11. Techniques and methods of VOCs analysis

There are several materials that can serve as a source for sampling, and they are called matrices. Human cadavers or tissue are included in primary matrices. However, in terms of gathering the scent samples, scientists choose mostly secondary matrices, like soil, air, or water, which have or had contact with decomposing remains (Stefanuto et al., 2017). Different variables in soil and air affect the types of VOCs received as a result. In soil, it might be the activity of the microbiota community, whereas in air VOCs tend to quickly disperse. In most studies, the process known as headspace sampling is employed as a starting point for VOCs collection. Headspace typically refers to the gaseous phase above the corpse. Despite the intensity of the decaying smell in the air, VOCs content is usually rather low (Forbes and Perrault, 2014). Taking it into account, stainless steel hoods above the carcasses (Stadler et al., 2013; Forbes and Perrault, 2014; Knobel et al., 2019), or body bags (Statheropoulos et al., 2005a,b, 2007, 2011) are often crucial step to increase the concentration of VOCs before sampling.

Nowadays, two concentration methods are at the forefront of decomposition VOCs research. First one – HS-SPME (headspace solid-phase microextraction) involves fused silica fiber, which is placed in a headspace above and remains for a specific amount of time so as to emitted VOCs may passively adsorb on a fiber coating. Subsequently captured VOCs undergo thermal desorption and are delivered into the gas chromatograph’s injection port for further analysis (Stefanuto et al., 2017). In the SPME method selection of optimal fiber coating may have an important role in getting the most complete profile of VOCs. Caraballo (2014) applied a standard mixture of VOCs previously identified during studies of odor mortis and found that for their extraction Carboxen/Polydimethylsiloxane (CAR/PDMS) fiber stood out in terms of sensitivity and selectivity (Caraballo, 2014).

Another approach is sorbent tubes, which can be used in either passive or dynamic ways. The static process is similar to SPME technique, since the sorbent tube located in a headspace captures VOCs on its adsorbent bed, while in active manner air is drawn through the tube additionally (Caraballo, 2014). Statheropoulos et al. (2005a,b) evaluated three VOCs sampling protocols with sorbent tube application. The first method included putting a Teflon tube ending with a sorbent tube in a hole of the body bag and using a pump to extract five liters of headspace. In the second method, the VOCs were initially placed in a 2 L flexible Tedlar bag employing the pump’s negative pressure to fill it before being transferred to the sorbent tube. During the third method, no pump was used and air from the cadaver bag diffuse directly into a sorbent tube. The initial approach produced the best results since it involved fewer steps where leaks may happen, and more air was tested. A dynamic way of sorbent tube usage has been shown to have an advantage over a passive one (Statheropoulos et al., 2005a,b). An interesting approach in terms of collecting VOCs was presented by DeGreeff and Furton (2011), who applied the Scent Transfer Unit – a field-portable headspace concentration tool. This device drew air, when it was swept above the body and the collecting material inside it concentrated the VOCs. In this study three sorbent materials were tested: Dukal cotton gauze, Johnson & Johnson cotton-blend gauze, and a polyester material. The aim was to find material, that would have enough affinity to analytes to bind them during collection but also allow to release them during desorption. Moreover, the optimal airflow rate was assessed too. When materials were used individually, the cotton-based fabrics yielded the highest amount of total compound, although with an increase in flow rate, the number of compounds visibly decreased in the case of Dukal Gauze. Since the polyester material demonstrated some potential in a matter of acids collection tough, the combination of sorbent materials was applied additionally. Overall, the Dukal gauze/polyester material at a low flow rate has been shown to be the most effective at recovering compounds (Perrault et al., 2015a,b,c). Some studies investigate collecting decomposition VOCs from the soil as well. The VOC-MoleTM soil probe is a common equipment for entrapping soil gas samples within the burial site. It is positioned in the ground, near the remains. A sorbent tube is attached to the probe’s cap. The air pump transfers air into the sampling tube from the soil probe (Forbes and Perrault, 2014; Perrault et al., 2015a,b,c).

The next step after collecting VOCs should be their separation and analysis. For such purposes, one-dimensional gas chromatography and mass spectrometry have been widely used in forensic studies over the years. Nowadays, thermal desorption coupled to two-dimensional gas chromatography–time-of-flight mass spectrometry (TD – GC × GC–TOFMS) seems to be a very promising analytical method. Firstly, TOFMS obtains entire mass spectra all at once, as opposed to scanning MS instruments, which read from high to low masses when the chromatographic peak is eluting. As a result, the gathered data are not affected by mass skewing (Agelopoulos and Pickett, 1998). This, in turn, guarantees the deconvolution of coeluting chromatographic peaks with distinct fragmentation patterns (Dallüge et al., 2003). If the peak apexes of coeluting substances are not less than three scans apart and have different mass spectra, the above property enables the identification of various compounds (Dekeirsschieter et al., 2012). Secondly, to accurately recreate elution currents, a rapid acquisition detector is necessary, because peak widths in the second dimension range from 50–400 ms. Therefore, TOFMS may be suitable for this purpose, since it has high acquisition rate capabilities (Dallüge et al., 2003). As a modulation method for TD -GC × GC–TOFMS cryomodulation is utilized far more commonly than flow modulation. Nevertheless, flow modulation seems to have an advantage over the initial process, according to Perrault et al. (2015a,b,c) this method may be an especially good approach for identifying ethers in the decomposition VOCs mixture. However, TD -GC × GC–TOFMS appeared to be unable to detect carboxylic acids.

Wisor et al., in their recent study, brought up an issue of difficulty in biogenic amines, such as putrescine and cadaverine, detection in decomposition odor. Due to primary amine properties (high basicity, high polarity, and low volatility), they are predisposed to be easily decomposed in GC capillary column, which impedes their analysis, and they are often undetectable in small quantities (Kataoka, 2005). Hence, finding an optimal method allowing the identification of all the amines produced during decomposition would be beneficial, especially when it comes to creating more precise HDR training tools. Taking it into account, Wisor (2021) proposed a derivatization process as a solution for transforming them into products more suitable for GC/MS analysis. After testing several chemical reactions to derivatize amines, the finest outcomes had been obtained with the production of carbamates using isobutyl chloroformate. Chromatogram of amines reacted with IBC had the most prominent amine peaks, which were well isolated from unconnected signals in comparison to other reactions. It was noted throughout this study that the proper adjustment of the amount of derivatization agent would be crucial in subsequent research because some of the amines, such as tyramine, may require different quantities to be visible on the chromatogram (Wisor, 2021).

## 12. Limitations and perspectives

Further investigations are necessary with the aim of standardization of human corpse odor profile, broadening the VOCs database, searching for human-specific biomarkers which could be targeted for efficient training for cadaver-detection dogs, and corroborating or clarifying studies results concerning the influence of external and internal factors on odor profile (Dekeirsschieter et al., 2009; Stefanuto et al., 2015; Forbes, 2017). In order to do so, it may be necessary to improve the sensitivity of methods detecting volatile chemical compounds released during the decomposition of the corpse, e.g., two-dimensional gas chromatography (Dekeirsschieter et al., 2009; Stefanuto et al., 2015). Likewise, elaborating portable detection devices and geophysical methods to track down buried human bodies would be remarkably helpful for the future of forensic disciplines (Rosier et al., 2015; Doro et al., 2022). Prospective studies of simulated disaster scenarios with shorter recovery periods seem to be promising for rescue personnel contributing to the fastest victim identification of the body before it reaches an advanced state of decay (that would hinder effective designation) (Perrault et al., 2015a,b,c). Cadaver-detection dogs should proceed to be trained on the scent of human remains, rather than porcine remains. Confirmation of the close similarity of the volatile chemical compounds released by both species during decomposition could tilt the balance in favor of using pig carcasses in the future (Raymer et al., 2020; Doro et al., 2022). The current statement regarding precedences in further forensic trials with the utilization of human remains suggests that matters which should be taken into consideration are including greater sampling sizes, longer collection timelines, and comparisons of approaches and locations between body farms (Titus et al., 2022). Volatile sulfur compounds are treated as one of the most biologically prominent VOCs released from humans during decomposition. Their detection (by GC-ICP-MS) has the potential to assist in forensic investigations and determining the time since death, but further research is needed to explore this issue with a more individualized approach and to supplement considerations of the effects of external factors (Clases et al., 2021). An important issue also seems to be a broader study of the odor profile during the decomposition of individual tissues, organs, and body fluids. Studies conducted on animal models (Cablk et al., 2012) clearly indicated the presence of significant differences in the classes of excreted VOCs between skin, muscle, and fat. It is necessary to transfer these studies to a human model, which would allow for improving the diagnosis of disputed forensic cases, where the only evidence is a body fragment. Of note in future research is the VOC profile depending on entomological diversity. Previous studies indicate the relationship between the presence of certain insect species and the presence of detectable concentrations of dimethyl trisulfide, dimethyl disulfide, phenol, and indole, which should be further characterized to develop forensic entomology.

To sum up, previous studies indicate the valuable role of VOC analysis in *postmortem* diagnostics. Thanks to the use of analytical chemistry methods, it is possible to determine the components of the odor emitted during decomposition and use them to determine the time and cause of death, train tracking dogs, and improve VOCs detection methods. Further studies are needed to discover the full VOC profile over time and environmental conditions.

## Author contributions

JC: Conceptualization, Visualization, Writing – original draft, Writing – review & editing. JS: Writing – original draft, Investigation. BN: Writing – original draft, Investigation. MK: Writing – original draft, Investigation. KM: Writing – original draft, Investigation. AP: Formal analysis, Supervision, Writing – review & editing. GJ: Formal analysis, Supervision, Writing – review & editing. MT: Conceptualization, Formal analysis, Funding acquisition, Project administration, Supervision, Writing – original draft, Writing – review & editing, Visualization.

## Funding

The author(s) declare financial support was received for the research, authorship, and/or publication of this article. This study was supported by Medical University of Silesia in Katowice. The authors would like to acknowledge funding from the National Science Foundation grants EES 2011764 and DUE 2151000.

## Conflict of interest

The authors declare that the research was conducted in the absence of any commercial or financial relationships that could be construed as a potential conflict of interest.

## Publisher’s note

All claims expressed in this article are solely those of the authors and do not necessarily represent those of their affiliated organizations, or those of the publisher, the editors and the reviewers. Any product that may be evaluated in this article, or claim that may be made by its manufacturer, is not guaranteed or endorsed by the publisher.
