# Supplementary material for: The smell of death. State-of-the-art and future research directions
Source: Front Microbiol. 2023 Sep 14;14:1260869. doi: 10.3389/fmicb.2023.1260869 (PMC10538644; doi:10.3389/fmicb.2023.1260869)
Supplement: Supplementary file 1 [file Table_1.docx]

| **Compound class** | **Chemical characterization of the class** | **Selected example volatile compounds** | **Decomposition phase** | **Approximate PMI** | **Extensive characterization** | **References** |
| --- | --- | --- | --- | --- | --- | --- |
| Cyclic hydrocarbons | Originated from lipids breakdown, in the headspace of blood clots mainly identified- aromatic hydrocarbons, for which ‘bloating’ is the most specific decomposition stage | toluene | anytime | 4 days- 2 years | Discovered in the headspace of adipose, muscle, skeletal, liver, kidney tissue, and in cremains samples | Titus et al., 2023; Vass et al., 2012; Perrault et al., 2015; Vass et al., 2008; Hoffman et al., 2009; Statheropoulos et al., 2005; Statheropoulos et al., 2007; Vass et al., 2004;  DeGreeff et al., 2011; Statheropoulos et al., 2006 |
|  |  | benzene | anytime | 5 years -10+ years | Identified in the breath of patients with lung cancer | Titus et al., 2023; Vass et al., 2012; Ioan et al., 2017; Perrault et al., 2015; Vass et al., 2008; Hoffman et al., 2009; Statheropoulos et al., 2005; Statheropoulos et al., 2007; Vass et al., 2004;  DeGreeff et al., 2011; Statheropoulos et al., 2006 |
|  |  | 1,2,4-trimethylbenzene | early | 5 -230 days | One of the most prominent volatile compounds during putrefaction | Titus et al., 2023; Moraleda et al., 2022; DeGreeff et al., 2011; Statheropoulos et al., 2006. |
|  |  | 1,2,3-trimethylbenzene | early | 5 -230 days | A specific compound for human remains VOCs profile | Titus et al., 2023; Perrault et al., 2015; Statheropoulos et al., 2005; Statheropoulos et al., 2007;  DeGreeff et al., 2011; Statheropoulos et al., 2006 |
|  |  | p-xylene | late | 4- 230 days | Tend to predominate in dry conditions during late stages of decay, found in decaying animal bones | Titus et al., 2023; Vass et al., 2012; Hoffman et al., 2009; Statheropoulos et al., 2005; Statheropoulos et al., 2007; Cablk et al., 2012; Statheropoulos et al., 2006 |
|  |  | styrene | early | 5 days-2 years | Usually likely to be detected at cIay/ loamy soil under anaerobic conditions | Vass et al., 2012; Vass et al., 2008; Statheropoulos et al., 2005; Statheropoulos et al., 2007; Vass et al., 2004; DeGreeff et al., 2011;, Statheropoulos et al., 2006 |
|  |  | naphthalene | anytime | 5 days-2 years | Can be detected in the expired air from fasting people | Vass et al., 2012; Ioan et al., 2017; Vass et al., 2008; Statheropoulos et al., 2005; Statheropoulos et al., 2007; DeGreeff et al., 2011; Statheropoulos et al., 2006 |
| Noncyclic hydrocarbons | Formed as a result of the breakdown of lipids, excreted from the body through the lungs or as a result of oxidation to alkyl alcohols by cytochrome P450, characteristic of the first day of "fresh decomposition" with increasing concentration during advanced decomposition | heptane | late | 30 days- 20+ years | Found in headspace air of bags with human bodies at the time of decay | Vass et al., 2012; Ioan et al., 2017; Perrault et al., 2015; Statheropoulos et al., 2005; Statheropoulos et al., 2007; Vass et al., 2004; Statheropoulos et al., 2006 |
|  |  | hexane | anytime | 5 days- 40+ years | One of the most prominent volatile compounds during putrefaction, found in decaying animal bones | Titus et al., 2023; Vass et al., 2008; Statheropoulos et al., 2005; Statheropoulos et al., 2007; Rosier et al., 2015; Cablk et al., 2012; DeGreeff et al., 2011; Statheropoulos et al., 2006 |
|  |  | 2-methylpentane | late | 16 days- 20+ years | Detected in the breath air of healthy people | Vass et al., 2012; Ioan et al., 2017; Perrault et al., 2015; Statheropoulos et al., 2005; Vass et al., 2004; Statheropoulos et al., 2006 |
|  |  | pentane | late | 16 days-40+ years | Seen later in the decomposition process in the dry soil | Vass et al., 2012; Perrault et al., 2015; Statheropoulos et al., 2005; Rosier et al., 2015;  DeGreeff et al., 2011; Statheropoulos et al., 2006 |
|  |  | octane | late | 16 days-40+ years | Tend to predominate in dry soil conditions during late stages of decay | Titus et al., 2023; Vass et al., 2012; Perrault et al., 2015; Statheropoulos et al., 2005; Statheropoulos et al., 2007; Statheropoulos et al., 2006 |
|  |  | undecane | early | 0-4 years | A specific compound for human remains VOCs profile | Titus et al., 2023; Vass et al., 2012; Statheropoulos et al., 2007; Rosier et al., 2015; Vass et al., 2004; DeGreeff et al., 2011; Statheropoulos et al., 2006 |
| Alcohols | Originated from carbohydrates, proteins, and lipids breakdown, an oxygen-free environment yielded the conversion of glucose monomers into alcohols cognate with butyric and acetic acids, many alcohols were identified from float, on the first day of ‘fresh stage ‘ alcohols are expected to increase, alcohols collected from the headspace of human tissue samples tend to decrease their abundance after day 13th, major components of freshly drawn blood | ethanol | late | 4 days – 40+ years | Bacterial species like *C. aminovalericum* and *C. cadaveris* are responsible for ethanol in the VOCs profile | Titus et al., 2023; Vass et al., 2012; Ioan et al., 2017; Vass et al., 2008; Statheropoulos et al., 2005; Statheropoulos et al., 2007; Rosier et al., 2015; Stefanuto et al., 2015; Vass et al., 2004; DeGreeff et al., 2011; Statheropoulos et al., 2006 |
|  |  | 1-butanol | early | 12 -230 days | Liberation of 1-Butanol after gasoline exposure, production related to the activity of *Bacillus subtilis* bacteria on the corpse | Titus et al., 2023; Ioan et al., 2017; Perrault et al., 2015; Statheropoulos et al., 2005; Rosier et al., 2015; Stefanuto et al., 2015; Cernosek et al., 2020; DeGreeff et al., 2011 |
|  |  | 1-hexanol, 2-ethyl | early | 5 -230 days | Detected in serum of hemodialysis patients and patients with liver cancer | Hoffman et al., 2009; Statheropoulos et al., 2005; Statheropoulos et al., 2007; Rosier et al., 2015; DeGreeff et al., 2011; Statheropoulos et al., 2006 |
|  |  | phenol | early | 5 -230 days | Treated as a common component of the human remains scent, high concentration in the expired air of fasting people, secretion associated with the activity of *Erysipelotrichaceae, Xanthomonadaceae*, and *Tissierellaceae* bacteria on corpses, a compound that attracts a number of worms | Titus et al., 2023; Vass et al., 2008; Statheropoulos et al., 2005; Statheropoulos et al., 2007;  Pascual et al., 2017; von Hoermann, 2016; DeGreeff et al., 2011 |
| Ketone/ ether | Originated from carbohydrates and lipids breakdown, lipases transform lipids from adipose tissue into e.g., unsaturated fatty acids and those sequentially toward ketones (in aerobic conditions), ‘bloating’ and ‘advanced decay’ are explicit for secretion ketones, major components of freshly drawn blood | 2-propanone (acetone) | late | 4days -40+ years | Formed by decarboxylation of acetoacetate, is perceived as the fruity aroma of old apple | Titus et al., 2023; Vass et al., 2012; Ioan et al., 2017; Vass et al., 2008; Moraleda et al., 2022; Javan et al., 2019; 26 Vass et al., 2004; DeGreeff et al., 2011; Statheropoulos et al., 2006 |
|  |  | 3-pentanone | early | 12/16-230 days | One of the most prominent volatile compounds during putrefaction | Titus et al., 2023; Perrault et al., 2015; Statheropoulos et al., 2005;  DeGreeff et al., 2011; Statheropoulos et al., 2006 |
|  |  | 2-pentanone | early | 12/16-230 days | Identified in the headspace air of bags containing the human corpse | Statheropoulos et al., 2005; DeGreeff et al., 2011; Statheropoulos et al., 2006 |
|  |  | 2-butanone | anytime | 4 days- 20+ years | Included in the potential “common core” of the VOCs profile identified during the decomposition process | Vass et al., 2012; Perrault et al., 2015; Moraleda et al., 2022; Javan et al., 2019;  DeGreeff et al., 2011; Statheropoulos et al., 2006 |
| Aldehydes | Originated from carbohydrates and lipids breakdown, muscle tissue decomposing predominantly into aldehydes, lipases transform lipids from adipose tissue into e.g., unsaturated fatty acids and those sequentially toward aldehydes (in aerobic conditions), tend to fluctuate over time of early decomposition | pentanal | anytime | 2-40+ years | - | Vass et al., 2012; Vass et al., 2008; Hoffman et al., 2009;  Statheropoulos et al., 2005; Vass et al., 2004; DeGreeff et al., 2011 |
|  |  | hexanaI | anytime | 2-10+ years | Investigated in the headspace of the human blood, adipocere and muscle tissue | Vass et al., 2012; Perrault et al., 2015; Vass et al., 2008; Hoffman et al., 2009;  42, DeGreeff et al., 2011; Statheropoulos et al., 2006 |
|  |  | nonanal | anytime | 20 days- 20+ years | Found in the air expired from fasting people | Vass et al., 2012; Perrault et al., 2015; Vass et al., 2008; Hoffman et al., 2009; Rosier et al., 2015; Vass et al., 2004; DeGreeff et al., 2011; Statheropoulos et al., 2006 |
|  |  | decanal | anytime | 23 days – 15+ years | Identified both in the Iiving and decaying human scent | Titus et al., 2023; Vass et al., 2012; Vass et al., 2008; Rosier et al., 2015;  Vass et al., 2004; DeGreeff et al., 2011; Statheropoulos et al., 2006 |
|  |  | benzaldehyde | anytime |  | Detectable in the blood of Iung cancer patients, production related to the activity of the bacteria *Ignatzschineria Ureiclastica* on the corpse | Perrault et al., 2015; Hoffman et al., 2009;, Rosier et al., 2015; Vass et al., 2004;  Pascual et al., 2017; DeGreeff et al., 2011 |
| Esters and acids | Volatile fatty acids are side products of breaking down muscle proteins and fat, oxygen-free environment yielded the conversion of glucose monomers to butyric and acetic acids, the most abundant class detected in human remains in winter trials. | hexadecanoic acid, methyl ester | early | 17 days- 230 days | Secretions related to the activity of *Tissierellaceae, Erysipelotrichaceae, Xanthomonadaceae,* and *Enterobacteriaceae* on cadavers | Vass et al., 2012; Vass et al., 2008; Statheropoulos et al., 2007; Vass et al., 2004;  Pascual et al., 2017; DeGreeff et al., 2011 |
|  |  | ethyl butyrate | early | - | Identified in the headspace air of bags containing the human corpse | Hoffman et al., 2009; Statheropoulos et al., 2005; Rosier et al., 2015; Statheropoulos et al., 2006 |
|  |  | ethyl acetate | early | - | May be connected with the plumes above the urban waste disposal bins | Ioan et al., 2017; Perrault et al., 2015; Statheropoulos et al., 2005; Rosier et al., 2015; Statheropoulos et al., 2006 |
|  |  | propyl acetate | early | - | Identified in the headspace air of bags containing human corpse | Statheropoulos et al., 2005; DeGreeff et al., 2011; Statheropoulos et al., 2006 |
| Sulfur compounds | Formed from microbial anaerobic breakdown of sulfur- containing amino acids (Met, Cys), the most evident class of VOCs during early stages of decomposition, diminish over time and tend to liberate most frequently in the ‘bloating’ and ‘active decay’ stages. | sulfur dioxide | early | 17 days -3 years | Characterized by unguent, irritating odor, found in decaying animal skin | Titus et al., 2023; Vass et al., 2012; Ioan et al., 2017; Perrault et al., 2015; Hoffman et al., 2009; Statheropoulos et al., 2005; Vass et al., 2004 |
|  |  | dimethyl disulfide | mid | 4 days -20+ years | Accumulate after amino acids desulfhydratation, has a strong disagreeable rotten egg-like, musty odor identified in the headspace of teeth has high vapor pressures – dissipates into the air before it is captured the most common product of mammalian decomposition, production related to the activity of *Bacillus subtilis* and *Ignatzschineria indica* bacteria on the corpse, a compound that attracts a number of worms | Titus et al., 2023; Vass et al., 2012; Ioan et al., 2017; Clases et al., 2021; Vass et al., 2008; Hoffman et al., 2009; Dekeirsschieter et al., 2009; Statheropoulos et al., 2005; Statheropoulos et al., 2007; Rosier et al., 2015; Stefanuto et al., 2015; Vass et al., 2004; Cernosek et al., 2020;  DeGreeff et al., 2011; Statheropoulos et al., 2006 |
|  |  | dimethyl trisulfide | early | 4 days- 2 years | Tend to accumulate near the corpse (rather than in the plume), a compound that attracts a number of worms | Titus et al., 2023; Vass et al., 2012; Ioan et al., 2017; Clases et al., 2021; Perrault et al., 2015; Vass et al., 2008; Dekeirsschieter et al., 2009; Statheropoulos et al., 2005; Statheropoulos et al., 2007; Rosier et al., 2015; Vass et al., 2004;, Zito et al., 2014; Trumbo et al., 2021  DeGreeff et al., 2011; Statheropoulos et al., 2006 |
|  |  | carbon disulfide | anytime | 16 days- 10+ years | - | Titus et al., 2023; Vass et al., 2012; Ioan et al., 2017; Perrault et al., 2015; Statheropoulos et al., 2005; Vass et al., 2004; DeGreeff et al., 2011; Statheropoulos et al., 2006 |
|  |  | dimethyl sulfide | late | 20+ years | Characterized by ‘halitosis’ like odor | Titus et al., 2023; Ioan et al., 2017; Statheropoulos et al., 2007; DeGreeff et al., 2011 |
|  |  | hydrogen sulfide | early | - | Characterized by a rotten egg-like odor, the dark coloration of the mottled skin during putrefaction is a sign of erythrocytes breakdown by bacterial enzymes, which is parallel to the liberation of hydrogen sulfide | Ioan et al., 2017; Perrault et al., 2015; Statheropoulos et al., 2005; Statheropoulos et al., 2007; DeGreeff et al., 2011 |
| Nitrogen compounds | Originated from proteins and nucleic acids breakdown, produced from the degradation of amino acids mainly due to Enterobacteriaceae enzymes, nitrogen-containing compounds increase over time and are most likely to be associated with the ‘active decay’ | methenamine | early | 23 days – 1,5 years | Identified during a simulated mass disaster event | Titus et al., 2023; Perrault et al., 2015; Vass et al., 2008; Hoffman et al., 2009; Statheropoulos et al., 2005; Statheropoulos et al., 2007; Vass et al., 2004; DeGreeff et al., 2011 |
| Halogen compounds | Originated from proteins (phosphorous), lipids, and nucleic acids breakdown, fluoride and chloride volatile compounds can derive from primary ingested and then absorbed by bones, blood, and soft tissue these elements | tetrachloroethene | anytime | 5 days- 5 years | - | Titus et al., 2023; Vass et al., 2008; Statheropoulos et al., 2005; Statheropoulos et al., 2007;  Vass et al., 2004; DeGreeff et al., 2011 |
|  |  | trichloromonofluoromethane | mid | 16 days -12 years | Identified during simulated mass disaster event | Vass et al., 2012; Perrault et al., 2015; Vass et al., 2004; DeGreeff et al., 2011 |
|  |  | chloroform | early | 16 days- 4 years /10+ years | Persist until all soft tissue is gone | Titus et al., 2023; Vass et al., 2012; Vass et al., 2008; Rosier et al., 2015; Vass et al., 2004; DeGreeff et al., 2011 |
|  |  | carbon tetrachloride | early | 0-3 years | Appears as a human remains-specific compound | Titus et al., 2023; Vass et al., 2008; Vass et al., 2004; DeGreeff et al., 2011 |

Table 1.
